# siRNA for Influenza Therapy

**DOI:** 10.3390/v2071448

**Published:** 2010-07-09

**Authors:** Sailen Barik

**Affiliations:** Department of Biochemistry and Molecular Biology, University of South Alabama, College of Medicine, MSB 2370, 307 University Boulevard, Mobile, AL 36688-0002, USA; E-Mail: sbarik@jaguar1.usouthal.edu; Tel.: +1-251-460-6860; Fax: +1-251-460-6850

**Keywords:** influenza virus, short interfering RNA, RNA interference, antiviral

## Abstract

Influenza virus is one of the most prevalent and ancient infections in humans. About a fifth of world’s population is infected by influenza virus annually, leading to high morbidity and mortality, particularly in infants, the elderly and the immunocompromised. In the US alone, influenza outbreaks lead to roughly 30,000 deaths each year. Current vaccines and anti-influenza drugs are of limited use due to high mutation rate of the virus and side effects. In recent years, RNA interference, triggered by synthetic short interfering RNA (siRNA), has rapidly evolved as a potent antiviral regimen. Properly designed siRNAs have been shown to function as potent inhibitors of influenza virus replication. The siRNAs outperform traditional small molecule antivirals in a number of areas, such as ease of design, modest cost, and fast turnaround. Although specificity and tissue delivery remain major bottlenecks in the clinical applications of RNAi in general, intranasal application of siRNA against respiratory viruses including, but not limited to influenza virus, has experienced significant success and optimism, which is reviewed here.

## 1. Introduction

Influenza virus is a widespread respiratory pathogen in humans and other animals, and is a major cause of morbidity and mortality globally [1–4]. Infection is acute, peaking roughly on the third day of exposure, and lasting about a week. There is a large variety of influenza viral strains, generated by a combination of mutations and re-assortment of genome segments [3,4]. This leads to antigenic shift and antigenic drift, turning all attempts of vaccine and antiviral design into a never-ending battle. Influenza virus strains have been isolated from divergent species such as chicken, duck, horse, and pig, and many have been shown to cross species boundaries through small mutational changes, raising the specter of a global epidemic. Regular influenza is a seasonal disease, suggesting its persistence in a natural reservoir that has not been clearly identified. New and continuously emerging viral strains, such as the recent pandemic swine-origin H1N1, evoke images of the historic 1918 “Spanish Flu” that killed about 50 million people, and infected 500 million, a third of the world’s population at the time [2,4]. Although small molecule antiviral regimens are available against influenza, they have limited efficacy and some are already rendered useless due to resistant viral mutations [1]. Given this bleak scenario, RNA interference (RNAi) offers a fresh and potentially effective antiviral strategy. In this mechanism, anti-influenza RNAi can be triggered by the use of synthetic small interfering RNAs (siRNAs) to promote sequence-specific degradation and silencing of the target viral RNA. In this article, I review the efforts in the anti-influenza siRNA area, analyze their future potential and summarize the challenges that lie ahead.

Other articles in this Issue cover various other antiviral approaches against influenza virus, including traditional and current ones, which will offer a more comprehensive perspective of our battle against this prehistoric pathogen.

## 2. Influenza and the Current Antiviral Therapy

Influenza, commonly called “flu”, is a highly contagious disease, and starts suddenly [3]. Common symptoms include high fever, headache, weakness, cough, sore throat, runny or stuffy nose, and body ache. In healthy adults, influenza is a self-limiting acute disease that lasts 7–10 days; however, it can be fatal in high-risk groups that include people 65 years or older, those with chronic medical conditions (such as asthma, diabetes, or heart disease) or with weak immunity (AIDS patients, pregnant women, and young children). Influenza can also invite opportunistic pathogens, leading to secondary infections such as bacterial pneumonia. The most common mode of horizontal spread of influenza virus (person to person) is via respiratory droplets in aerosols produced by coughing or sneezing of infected people. Transmission of influenza virus and other respiratory viruses also occurs by touching contaminated objects (such as door knobs) followed by touching the nose or the eyes [5].

Influenza viruses are members of the *Orthomyxoviridae* family, and the viral genome consists of eight negative sense (anti-mRNA sense) RNA segments [3]. Together, these segments code for 10 distinct viral proteins: three subunits of the viral RNA-dependent RNA polymerase (RdRP) (PA, PB1, PB2), hemagglutinin (HA) and neuraminidase (NA) that are major surface glycoproteins, nucleocapsid protein (NP), matrix proteins (M1, M2) and nonstructural proteins NS1 and NS2. All these gene products are indispensable for optimal virus replication in cell culture and/ or animal hosts. The genomic viral RNA (vRNA) is wrapped with NP protein and the resultant ribonucleoprotein complex is specifically transcribed by the viral RdRP to produces mRNA with 5′-cap and the 3′-poly A tail, which serves as a template for viral proteins synthesis. Copying of the vRNA also produces complementary RNA (cRNA) lacking the 5′-cap and the 3′-poly A tail; being positive-sense and lacking translatability, the cRNA only serves as a template for more vRNA. Based on the sequence diversity of the NP and M proteins, the influenza viruses are currently classified into three types: A, B, or C.

Although all influenza viruses can cause morbidity and mortality, the influenza A viruses have caused pandemic outbreaks such as those in 1918, 1957, 1968 and now in 2009 with the swine-origin H1N1 influenza virus [3,4]. Two of the viral proteins, neuraminidase (NA) and the M2 ion channel protein, are the targets for the US Food and Drug Administration (FDA)-approved influenza antiviral drugs. While oseltamivir (Tamiflu) and zanamivir (Relenza) are NA inhibitors, amantadine (Symmetrel) and rimantadine inhibit M2 [1]. Unfortunately, there is now widespread viral resistance to both drug classes, but particularly to the M2 inhibitors. Mutagenesis is the most important cause of viral resistance, primarily due to the error-prone copying of the RNA genome by viral RdRP and the ability of the virus to reassort its genome segments [1]. With the limited number of viral drug targets for influenza virus, this creates concern for the development of new influenza therapies.

## 3. siRNA as Antiviral: an Historical Perspective

RNAi is a recently discovered regulatory pathway that is initiated by double-stranded RNA [6,7]. RNAi works by a dice-and-slice mechanism in which long dsRNA is first cleaved by a specialized RNase, named Dicer, to generate 20–22 base-pair products with two-nucleotide long 3′-overhangs. These Dicer products are commonly known as short interfering RNA or siRNA. The antisense strand of the siRNA is then recruited into the RNA-induced silencing complex (RISC), the key catalytic component of which is the “slicer” RNase, Argonaute 2 (Ago2). Using the antisense RNA strand as a ‘guide’, the RISC engages the complementary target mRNA, which is then sliced and destroyed by Ago2, leading to silencing of expression of the cognate gene. However, the use of RNAi as a gene silencing tool really prospered after the demonstration in 2001 that chemically synthesized siRNA, when transfected into mammalian cells in culture, simulates natural RNAi and specifically destroys the complementary target mRNA [8]. The first proof of concept that synthetic siRNA can be antiviral also appeared in 2001 [9], whereby growth of respiratory syncytial virus (RSV) was inhibited by targeting essential viral mRNAs coding for the viral transcription factor, P (phosphoprotein) and viral F (fusion) protein. In both cases, specific ablation of the corresponding protein and mRNA was demonstrated. Moreover, it was also shown that while the mRNAs of RSV were vulnerable to siRNA, the viral genome in contrast was resistant, likely due to the fact that the negative strand RNA viral genome is generally wrapped with the viral nucleocapsid protein (N or NP) and hence inaccessible to the guide strand RNA [9]. Since then, the siRNA-based approach has been applied with success against all major acute viral infections, including the first report in influenza by the Chen laboratory in 2003 [10]. These and other results are detailed below.

## 4. Inhibition of Influenza by siRNA Targeting Viral mRNA

Due to their small genome, virtually all RNA viral genes serve an essential role in virus replication. Conceptually, silencing of any of them will inhibit virus growth to some extent. This has been borne out by the large body of accumulated evidence in a variety of viruses [11]. As mentioned, the high mutability of RNA viral genomes in general and the reassortment of genome segments in segmented viruses have generated many strains and subtypes of influenza virus. The influenza A virus, for example, boasts 15 HA and nine NA subtypes. An effective strategy of siRNA design is generally based on the following considerations. First of all, as the genome RNA is encapsidated by the N (NP) protein, siRNA should target the mRNAs instead of the genome [9]. Second, in order to design siRNAs that will remain effective despite sequence changes over time, the most conserved sequences of a given mRNA should be targeted. For influenza virus, a battery of siRNAs specific for NP, PA, PB1, PB2, M, and NS genes were designed and tested with various degrees of inhibition of multiple subtypes [10]. For example, in mouse experiments [12,13], the same anti-NP and anti-PA siRNA inhibited the growth of all of the following strains and subtypes, with inhibition ranging from 9 to 56 fold: A/Puerto Rico/8/34 (H1N1), A/Hong Kong/156/97 (H5N1), A/ Netherlands /219/03 (H7N7), and A/Hong Kong/1073/99 (Avian H9N2). In contrast, it was not possible to design such broad-spectrum siRNAs against the more divergent HA and NA sequences because no stretch of 21 conserved nucleotides could be found in these sequences [10], which also underscores the reason for rapid resistance against current drugs that target the neuraminidase. A list of published siRNA sequences targeting influenza viral mRNAs [10] are shown in Table 1.

## 5. Inhibition of Influenza by siRNA Targeting Cellular mRNA

As obligatory parasites, all viruses depend on cellular functions for productive infection, and influenza virus is no exception [14]. Thus, an alternative and complementary antiviral strategy would be based on knocking down these cellular functions to inhibit virus growth and hence the associated pathology. This concept has recently been validated for small molecule inhibitors. For example, chemical inhibitors of the cellular ErbB-1 signaling pathway (which facilitates poxvirus replication) prevented Vaccinia and Variola virus infection [15]. Another example is to target cellular receptors, which blocks viral infection at the earliest stage. Indeed, small molecules that bind to the HIV coreceptors (CXCR4, CCR5) have shown promise as effective HIV antivirals [16].

In recent years, genome-wide high-throughput siRNA screening was performed, first in surrogate *Drosophila* cells [17] and then in A549 cells of human origin [18,19], to identify cellular genes needed for influenza virus growth. The gene sets discovered by different groups showed substantial commonality, and many were validated by secondary knockdowns. Together, these studies revealed essential roles of dozens of cellular genes in influenza replication. A few notable ones include: SON DNA binding protein (SON), CDC-like kinase 1 (CLK1), vacuolar ATPase (ATP6V0D1), COPI coated vesicle transport (COPG), eIF4A3, fibroblast growth factor receptor (FGFR) proteins, and glycogen synthase kinase 3 (GSK3)-β, nuclear import components, proteases, and the calcium/calmodulin-dependent protein kinase (CaM kinase) IIβ (CAMK2B). The reliability of the approach was evidenced in part by the rediscovery of components previously known to be important for influenza growth, such as nuclear export factors NXF1 [20] and XPO1 [21]. Validated siRNAs against these and other cellular genes can be purchased from multiple commercial sources, which could expedite their further development as anti-influenza drugs in animal studies followed by clinical trials.

As the cellular genes perform important functions in the normal cell, potential side-effect of these drugs or siRNAs is a genuine concern. However, some gene products may be partially dispensable, at least for a short term. For example, siRNA-based silencing of cellular genes revealed that the actin-regulatory proteins, VASP (Vasodilator-stimulated phosphoprotein) and Zyxin, are dispensable for the growth of cultured cells [22]. The dispensability is further validated by the fact that VASP and Zyxin knockout mice are fully viable and fertile, with virtually no phenotype [23,24]. Interestingly, both proteins, as also another actin-regulatory protein, profilin, were found to be essential for the morphogenesis of respiratory syncytial virus, such that knockdown of VASP, Zyxin, or profilin by siRNA strongly inhibits progeny virus synthesis [25–28]. Together, these results provide proof-of-concept that some virally essential cellular functions can be less essential for the cell itself, and thus, can be targeted by antivirals, at least for the short time frame of an acute respiratory infection, such as influenza.

## 6. Testing Anti-influenza siRNA

As a rule, the anti-influenza potency of a synthetic siRNA is first tested in cultured cells by using a series of siRNA concentrations. The Madin-Darby Canine Kidney (MDCK) cell line, commonly used for routine influenza virus growth, is well suited for this purpose [10,29]. As mentioned above, the human alveolar epithelial carcinoma A549 cell line has also been used for its human relevance, particularly for cellular gene knockdown studies using human genome-specific siRNAs [18,19]. In cell culture studies, live virus infection can be substituted by transfected influenza-based reporter plasmids, expressing enzymes such as Luciferase, amenable to high-throughput screening [29]. For testing on a smaller scale, egg yolk sac (in 10-day embryonated chicken eggs) is also used, and the liberated virus in allantoic fluid is harvested and assayed. Influenza virus titer can be measured by routine hemagglutination or plaque assay [29], or the less commonly used immunofluorescence imaging, or luciferase assays using a reporter plasmid in which the luciferase gene is transcribed from an influenza viral promoter, thus requiring virus infection [19]. In general, results from these different screening platforms have been highly corroborative, *i.e.,* siRNAs that strongly inhibited influenza virus production in cultured cells also inhibited virus production in chicken embryos [10,12,13,30].

## 7. Anti-influenza siRNA Testing in an Animal Model

The siRNA that show potency against influenza virus in cell culture is then tested in the mouse model. In one the earliest reports [12], C57BL/6 black mice were used, and the synthetic siRNAs, complexed with the cationic polymer polyethyleneimine (PEI), were administered intravenously (IV). In another study [13], siRNA was complexed with Oligofectamine (Invitrogen) administered and applied intranasally (IN). Significant reduction of influenza titer in the animal lung as well as relief of symptoms was observed in both cases. The siRNAs were target-specific, did not activate interferon, and were effective in both prophylaxis and therapy. Moreover, influenza-specific siRNA treatment was broadly effective and protected animals against lethal challenge with highly pathogenic avian influenza A viruses of the H5 and H7 subtypes [13].

The IN route is particularly promising for the prevention and therapy of influenza and other respiratory viral infections [13,31,32] since it delivers the siRNA directly to the lung, the primary site of infection, thus ensuring the specificity of delivery and minimizing systemic siRNA loss and toxicity (see below).

## 8. Advantages of siRNA as Anti-influenza Drug

The siRNA-based antiviral approach has the following advantages of traditional antiviral compounds: (i) It is a highly potent antiviral agent with preventive as well as therapeutic value; (ii) It can be designed and synthesized in hours, and new ones can be rapidly deployed if resistance to the first set is encountered; (iii) It can be applied in combination with other siRNAs in a multidrug regimen to reduce the odds of resistance or to target multiple co-infecting viruses; (iv) If used IN, it is noninvasive and can be readily administered via standard hand-held inhalers; (v) Dry siRNA in powder form is relatively stable and can be transported without refrigeration to remote locations and extreme climates; when needed, the influenza patient can just add water to it, mix and use as nasal drops or inhaled aerosol. Together, intranasal anti-influenza siRNA can match the virus in speed, tissue specificity, and broad-spectrum potency.

As in any therapeutic regimen, however, intranasal applications need to be optimized for potential side effects. The previously mentioned Zanamivir, as sold under the name ‘Relenza’ by GlaxoSmithKline, is applied via a hand-held nasal inhaler and is generally well-tolerated. However, its side-effects may include exasperation of asthma, bronchospasm, coughing, dizziness, headache, nausea, sinus inflammation, sore throat, stuffy nose and facial swelling, although the exact mechanism of these reactions remains unclear.

## 9. Conclusion: The Future of Anti-influenza siRNA

The path of the pharmaceutical development of anti-influenza siRNA shares the general hurdles that confronts all siRNA.

First, there are reports that siRNA may trigger innate immune reactions, such as the Toll-Like Receptor (TLR) pathway, leading to inflammation and shock [33–35]. However, it appears that the extent of such nonspecific reactions varies among cell types, siRNA sequences and transfection reagents. We and others have shown that optimization of these parameters can indeed lead to safe and effective siRNA [9,12,13,31,36]. For example, the motifs 5′-GUCCUUCAA-3′ [33] and 5′-UGUGU-3′ [34] have been identified to be immunostimulatory and hence avoided in designing siRNA sequences. In our hands, PEI almost invariably killed the mice when administered IN, whereas other reagents, including Oligofectamine [13], and Transit-KO (Mirus) [31] posed no problem.

Second, stability of the siRNA in biological fluids remains a concern, although IN application, such as against RSV, SARS coronavirus, parainfluenza and influenza, appears to largely bypass this problem [12,13,31,37].

To address these concerns, various chemical modifications in the siRNA, such as 2’-O-methyl and 2’-fluoride substitutions in the ribose ring have been tested, which retained specificity and potency without causing untoward reactions [38]. In addition, novel second generation transfection reagents [39] and nanoparticle technology [40] are being developed to bypass the TLR pathway, which have shown promising results.

In an interesting novel modality, immunostimulation by siRNA may actually be harnessed for its broad-spectrum antiviral effect. In a recent study [41], a non-pairing uridine bulge in the ‘passenger’ strand (complementary to the ‘guide’ strand) was introduced in a siRNA against Semliki Forest virus (SFV), such that the modified siRNA resembled the micro-RNA (miRNA) family of short RNA, naturally found in normal cells. The sequence modification did not reduce the silencing activity of the siRNA but increased proinflammatory cytokine production, resulting in enhanced protection against SFV. In another study [42], the residual part of the mRNA following siRNA-mediated cleavage was shown to translate shorter, N-terminal protein fragments; when the cleavage was directed downstream of a cytotoxic T-cell epitope, the shorter fragments that contained the epitope more generated protective immune response. Both these recent approaches can be easily tested and adopted for influenza viral targets. In summary, there is significant optimism that an anti-influenza siRNA drug may be in clinical trial in short order.

## Figures and Tables

**Table 1:** Validated siRNA sequences targeting influenza viral mRNAs [10].

**Target viral mRNA (nt #)**	**siRNA sequence: top strand 5′ to 3′**
PB2-2210	GGAGACGUGGUGUUGGUAAdTdT dTdTCCUCUGCACCACAACCAUU
PB2-2240	CGGGACUCUAGCAUACUUAdTdT dTdTGCCCUGAGAUCGUAUGAAU
PB1-6	GCAGGCAAACCAUUUGAAUdTdT dTdTCGUCCGUUUGGUAAACUUA
PB1-129	CAGGAUACACCAUGGAUACdTdT dTdTGUCCUAUGUGGUACCUAUG
PB1-2257	GAUCUGUUCCACCAUUGAAdTdT dTdTCUAGACAAGGUGGUAACUU
PA-44	UGCUUCAAUCCGAUGAUUGdTdT dTdTACGAAGUUAGGCUACUAAC
PA-739	CGGCUACAUUGAGGGCAAGdTdT dTdTGCCGAUGUAACUCCCGUUC
PA-2087	GCAAUUGAGGAGUGCCUGAdTdT dTdTCGUUAACUCCUCACGGACU
PA-2110	UGAUCCCUGGGUUUUGCUUdTdT dTdTACUAGGGACCCAAAACGAA
PA-2131	UGCUUCUUGGUUCAACUCCdTdT dTdTACGAAGAACCAAGUUGAGG
NP-231	UAGAGAGAAUGGUGCUCUCdTdT dTdTAUCUCUCUUACCACGAGAG
NP-390	UAAGGCGAAUCUGGCGCCAdTdT dTdTAUUCCGCUUAGACCGCGGU
NP-1496	GGAUCUUAUUUCUUCGGAGdTdT dTdTCCUAGAAUAAAGAAGCCUC
M-37	CCGAGGUCGAAACGUACGUdTdT dTdTGGCUCCAGCUUUGCAUGCA
M-480	CAGAUUGCUGACUCCCAGCdTdT dTdTGUCUAACGACUGAGGGUCG
M-598	UGGCUGGAUCGAGUGAGCAdTdT dTdTACCGACCUAGCUCACUCGU
M-934	GAAUAUCGAAAGGAACAGCdTdT dTdTCUUAUAGCUUUCCUUGUCG
NS-128	CGGCUUCGCCGAGAUCAGAdAdT dTdAGCCGAAGCGGCUCUAGUCU
NS-562	GUCCUCCGAUGAGGACUCCdTdT dTdTCAGGAGGCUACUCCUGAGG
NS-589	UGAUAACACAGUUCGAGUCdTdT dTdTACUAUUGUGUCAAGCUCAG
